# Marine algal flora of Graciosa Island, Azores

**DOI:** 10.3897/BDJ.8.e57201

**Published:** 2020-10-02

**Authors:** Ana I. Azevedo Neto, Manuela I. Parente, Andrea Z. Botelho, Afonso C. L. Prestes, Roberto Resendes, Pedro Afonso, Nuno V. Álvaro, David Milla-Figueras, Raul M. A. Neto, Ian Tittley, Ignacio Moreu

**Affiliations:** 1 cE3c - Centre for Ecology, Evolution and Environmental Changes/Azorean Biodiversity Group & Faculdade de Ciências e Tecnologia, Departamento de Biologia, Universidade dos Açores, 9500-321 Ponta Delgada, São Miguel, Açores, Portugal cE3c - Centre for Ecology, Evolution and Environmental Changes/Azorean Biodiversity Group & Faculdade de Ciências e Tecnologia, Departamento de Biologia, Universidade dos Açores 9500-321 Ponta Delgada, São Miguel, Açores Portugal; 2 CIBIO, Centro de Investigação em Biodiversidade e Recursos Genéticos, InBIO Laboratório Associado, Pólo dos Açores, Universidade dos Açores, 9500-321 Ponta Delgada, São Miguel, Açores, Portugal CIBIO, Centro de Investigação em Biodiversidade e Recursos Genéticos, InBIO Laboratório Associado, Pólo dos Açores, Universidade dos Açores 9500-321 Ponta Delgada, São Miguel, Açores Portugal; 3 Universidade dos Açores, Faculdade de Ciências e Tecnologia, Departamento de Biologia, 9500-321 Ponta Delgada, São Miguel, Açores, Portugal Universidade dos Açores, Faculdade de Ciências e Tecnologia, Departamento de Biologia 9500-321 Ponta Delgada, São Miguel, Açores Portugal; 4 IMAR/Okeanos – University of the Azores, Departamento de Oceanografia e Pescas, Universidade dos Açores, Rua Prof. Doutor Frederico Machado, 9901-862 Horta, Faial, Portugal IMAR/Okeanos – University of the Azores, Departamento de Oceanografia e Pescas, Universidade dos Açores, Rua Prof. Doutor Frederico Machado 9901-862 Horta, Faial Portugal; 5 Universidade dos Açores, Faculdade de Ciências Agrárias, CCMMG (Centro do Clima Meteorologia e Mudanças Globais), IITA-A (Instituto de Investigação e Tecnologias Agrárias e do Ambiente), 9700-042 Angra dp Heroísmo, Terceira, Portugal Universidade dos Açores, Faculdade de Ciências Agrárias, CCMMG (Centro do Clima Meteorologia e Mudanças Globais), IITA-A (Instituto de Investigação e Tecnologias Agrárias e do Ambiente) 9700-042 Angra dp Heroísmo, Terceira Portugal; 6 N/A, Odivelas, Portugal N/A Odivelas Portugal; 7 Natural History Museum, Cromwell Road, London, United Kingdom Natural History Museum, Cromwell Road London United Kingdom

**Keywords:** Macroalgae, Azores, Graciosa Island, new records, endemism, native, uncertain, introduced, occurrence data.

## Abstract

**Background:**

The macroalgal flora of Graciosa (central group of Azores archipelago) is poorly known, with only 116 species recorded so far (authors personal data). The published information reflects occasional collections from sporadic field visits to the Island. To overcome this, a thorough investigation under the Expedition “GRACIOSA/2004”, the Campaigns “PADEL/2006”, “MACROBIOLMOL/2014” and “PIMA-BALA/2017” involving sample collecting and presence data recording, was undertaken over an area of 19 km^2^ encompassing littoral and sublittoral levels down to about 40 m around the Island. This paper lists the taxonomic records and provides information on species ecology and occurrence around the Island improving the knowledge of the Azorean macroalgal flora at both local and regional scales.

**New information:**

A total of 1692 specimens belonging to 250 taxa of macroalgae (and including 55 taxa identified only at the genus level) are registered, comprising 166 Rhodophyta, 36 Chlorophyta and 48 Ochrophyta (Phaeophyceae). From these, 195 are identified to the species level (126 Rhodophyta, 31 Chlorophyta and 38 Ochrophyta) and comprise 156 native, 20 of uncertain origin and 14 introductions to the Island. Predaea feldmannii subsp. azorica Gabriel is an Azorean endemic, whereas *Codium elisabethiae* O.C. Schmidt, *Botryocladia macaronesica* Afonso-Carrillo, Sobrino, Tittley & Neto, *Phyllophora gelidioides* P.Crouan & H.Crouan ex Karsakoff and *Laurencia viridis* Gil-Rodríguez & Haroun represent Macaronesian endemics. Seventy-nine species are newly recorded to the algal flora of the Island.

## Introduction

Around 400 species of marine macroalgae have been recorded in the isolated mid-Atlantic Azores archipelago so far (Freitas et al. 2019). The Azorean algal flora is cosmopolitan with species shared with Macaronesia *sensu lacto*, North Africa, the Mediterranean Sea, Atlantic Europe and America. Overall, it shares more species with the east Atlantic flora than with the west (Tittley and Neto 2006, Wallenstein et al. 2009).

Based on extensive analysis encompassing widely-dispersing phyla (as coastal fishes, echinoderms and macroalgae) and less-dispersing phyla (as brachyurans, polychaetes and gastropods), Freitas et al. (2019) suggested that the Azores should be a biogeographical entity of its own and proposed a re-definition of the Lusitanian biogeographical province, in which they consider four ecoregions: the South European Atlantic Shelf, the Saharan Upwelling, the Azores ecoregion and a new ecoregion herein named Webbnesia, which comprises the archipelagos of Madeira, Selvagens and the Canary Islands. In their paper, when comparing the Azorean algal flora to that of the new Webbnesia region, they reported that the Canary Islands, with 689 species, are by far the most diverse archipelago, followed by the Azores (405), Madeira (396) and Cabo Verde (333). The Selvagens are the least diverse one (295 species). When compared to that of other remote oceanic Islands (e.g. the Shetlands and Faroes in the colder North Atlantic and Ascension and Tristan da Cunha in the Southern Atlantic), the algal flora of the Azores can also be considered relatively rich (Tittley 2003, Neto et al. 2005, Tittley and Neto 2005, Tittley and Neto 2006, Wallenstein et al. 2009).

From all Azorean Islands, São Miguel is by far the one with the largest amount of research dedicated to the study of the algal flora. The total number of algal species is at the moment 260, a number that is likely to increase due to ongoing research by authors of the present paper. Most of the remaining Islands have received less attention. To overcome this and to improve the understanding of the archipelago’s macroalgal flora, research has been conducted over the past three decades in all the Islands. Data on the Islands of Pico and Terceira are already available on the recently-published papers by Neto et al. (2020a), Neto et al. (2020b). Table 1summarises the available information at the moment.

The present paper presents both physical and occurrence data and information gathered from macroalgae surveys undertaken on Graciosa (central group of the archipelago) by the Island Aquatic Research Group of the Azorean Biodiversity Centre of the University of the Azores (Link: https://ce3c.ciencias.ulisboa.pt/sub-team/island-aquatic-ecology), the MARBE, Marine Biodiversity and Environment Research Group of CIBIO-Açores at the University of the Azores (Link: http://cibio.uac.pt/en/research-groups/marbe-marine-biodiversity-and-environment) and the OKEANOS Centre of the University of the Azores (Link: http://www.okeanos.uac.pt). In these surveys, particular attention was given to the small filamentous and thin sheet-like forms that are often short-lived and fast-growing species, very difficult to identify in the wild, requiring the aid of a microscope.

The paper aims to provide a practical resource for biological studies, such as systematics, diversity and conservation, biological monitoring, climate change and ecology and also for academics, students, government, private organisations and the general public.

## General description

### Purpose

In this contribution, we list taxonomic records for Graciosa and present general information for the occurrence of each taxon around the Island. By doing this, we are contributing to address several biodiversity shortfalls (see Cardoso et al. 2011, Hortal et al. 2015), namely, the need to catalogue the Azorean macroalgae (Linnean shortfall) and to improve the current information on their local and regional geographic distribution (Wallacean shortfall), as well as on species abundances and dynamics in space (Prestonian shortfall).

## Project description

### Title

Marine algal flora of Graciosa Island, Azores

### Personnel

Collections were undertaken and occurrence data recorded during several years (2004-2017). The main collectors were Amine Sebti, Ana F. Ferreira, Ana I. Neto, André Amaral, Andrea Z. Botelho, Catarina Santos, Daniel Torrão, Daniela Gabriel, David Milla-Figueras, Edgar Rosas-Alquicira, Eunice Nogueira, Francisco Wallenstein, Gonçalo Graça, Inês Machado, João Brum, Jorge Fontes, José M. N. Azevedo, Ian Tittley, Karla Leon Cisneros, Manuela I. Parente, Marlene Terra, Nil Alvaréz-Segura, Nuno Álvaro, Pedro Monteiro, Pedro Raposeiro, Patrícia Madeira, Raquel Torres, Ruben Couto, Rui Sousa, Sara Peres and Sandra Medeiros.

Preliminary *in situ* identifications were done by Ana I. Neto, Andrea Z. Botelho, Daniela Gabriel, David Milla-Figueras, Edgar Rosas-Alquicira, Francisco Wallenstein, Karla Leon Cisneros, Ian Tittley, Manuela I. Parente, Marlene Terra and Ruben Couto.

Ana I. Neto, Andrea Z. Botelho, David Milla-Figueras, Edgar Rosas-Alquicira, Karla Leon Cisneros, Ian Tittley and Manuela I. Parente were responsible for the final species identification.

Voucher specimen management was mainly done by Afonso Prestes, Ana I. Neto, Andrea Z. Botelho, David Milla-Figueras, Eunice Nogueira, Manuela I. Parente, Natália Cabral and Roberto Resendes.

### Study area description

**Figure 1.**
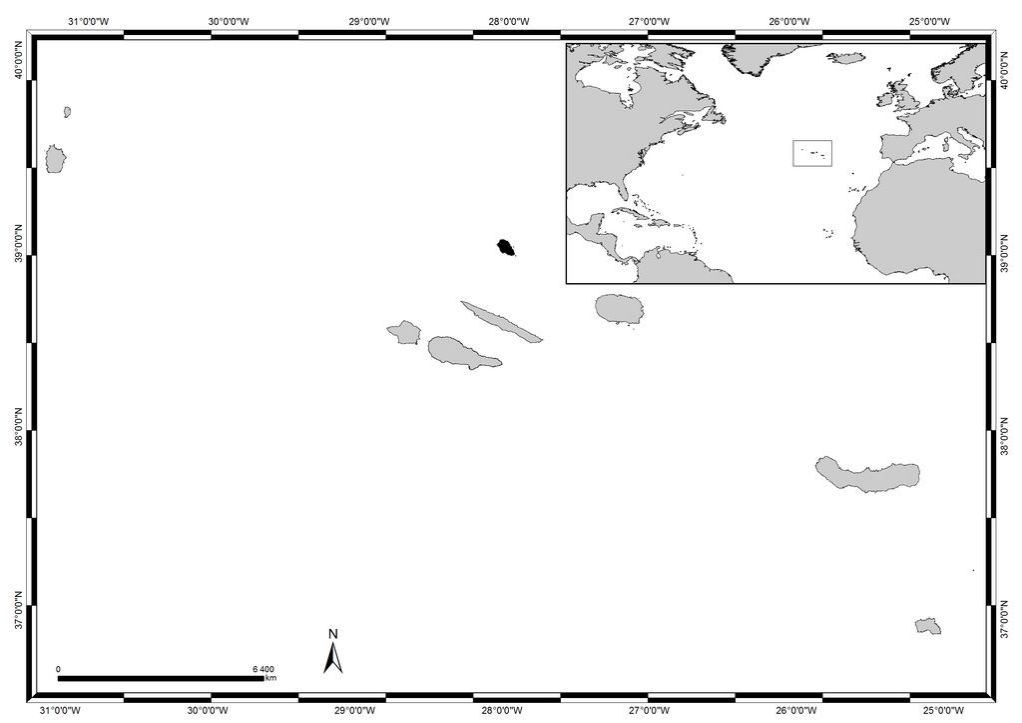
The Azores, its location in the Atlantic and Graciosa Island highlighted in black (by Nuno V. Álvaro).

The Azores archipelago, located in the North Atlantic, roughly at 38°43′49″N 27°19′10″W (Fig. 1) comprises nine Islands and several Islets spread over 500 km in a WNW direction. The climate is temperate oceanic, with persistent winds, regular and abundant rainfall and high levels of relative humidity mainly during winter and autumn (Morton et al. 1998). The Islands have a restricted coastal extension due to the lack of a continental shelf and deep waters occur within a few kilometres offshore. The tidal range is small (< 2 m, see Hidrográfico 1981) and coasts are subjected to swell and surge most of the year. Shore geomorphology alternates between high cliffs and rocky cobble/boulder beaches (Borges 2004).

Graciosa (in black in Fig. 1) is the second smallest Island of the Azores archipelago. Located in the central group, roughly at 39°0′38″N, 27°59′1″W, about 37 km north of São Jorge and 58 km north-west of Terceira, it has an area of about 62 km^2^ and a maximum altitude of 402 m at the summit of the Caldeira, located at the south-western tip of the Island (Neto et al. 2009).

With the exception of Serra Branca, bordered by cliffs higher than 200 m and the area between Lagoa and Barra, where the coastline consists of steep cliffs, the remaining coastline of the Island is low (below 50 m), with long stretches of cobble beaches interspaced with lava flows (forming irregular extensions of bedrock), boulder areas and the single sandy beach near the small village of Praia. Between Ponta Branca and Carapacho, there are several bays, of which the bay of Filipe is the largest in size and easily accessible by land. Rock pools are common on the bedrock shores around the Island, creating a shallow subtidal habitat with a rich diversity of marine life (Neto et al. 2009).

**Figure 2.**
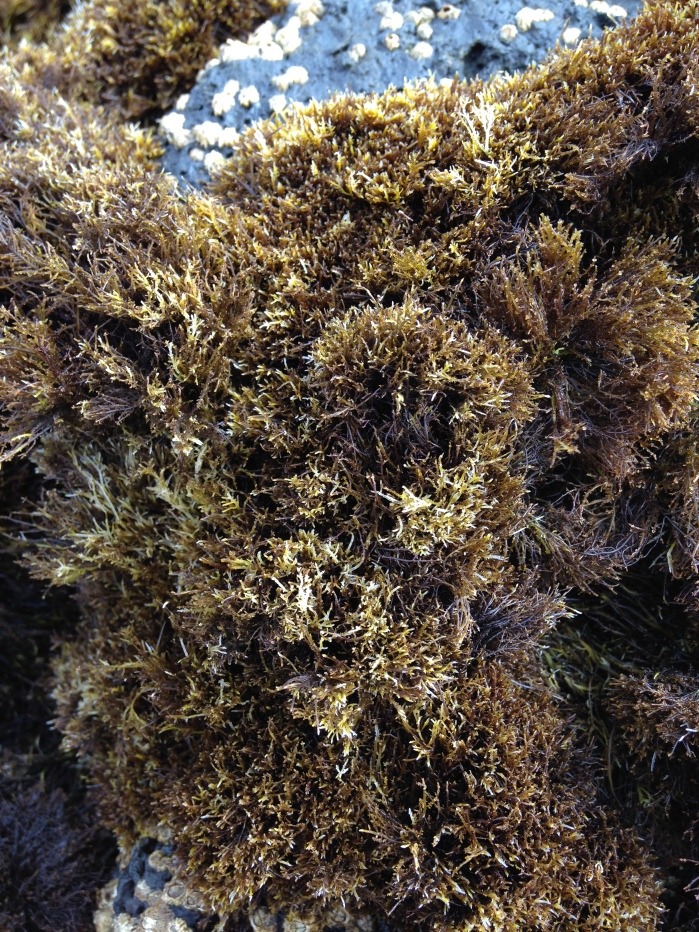
The red macrophyte *Gelidium microdon*, a characteristic species of the Azorean high intertidal level (by the Island Aquatic Ecology Subgroup of cE3c-ABG).

**Figure 3.**
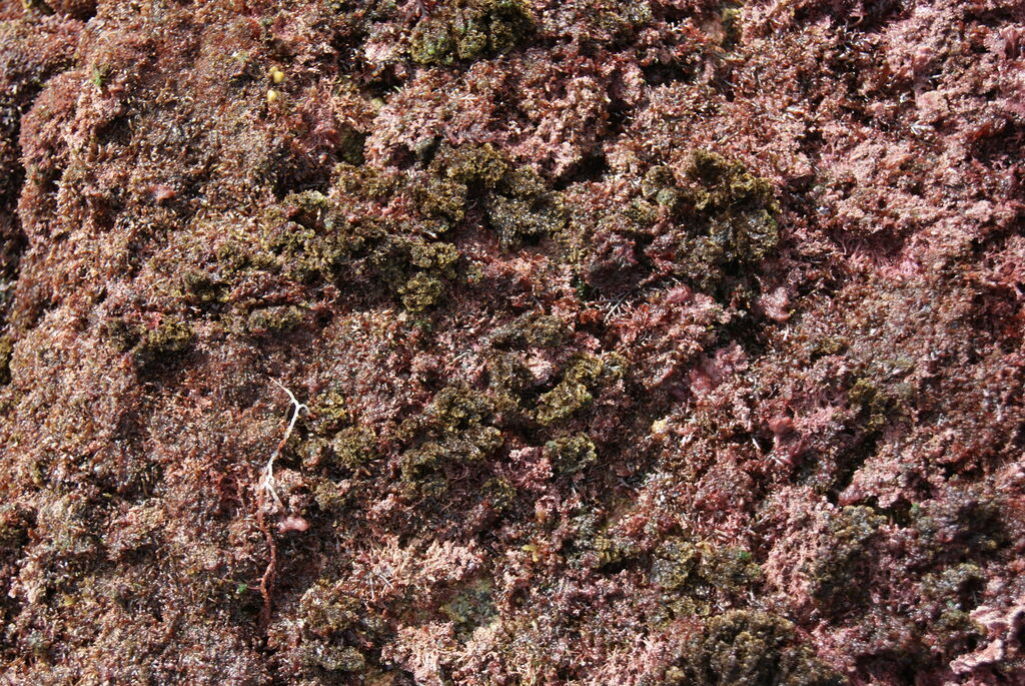
Algal turf at mid-shore intertidal level (by the Island Aquatic Ecology Subgroup of cE3c-ABG).

**Figure 4.**
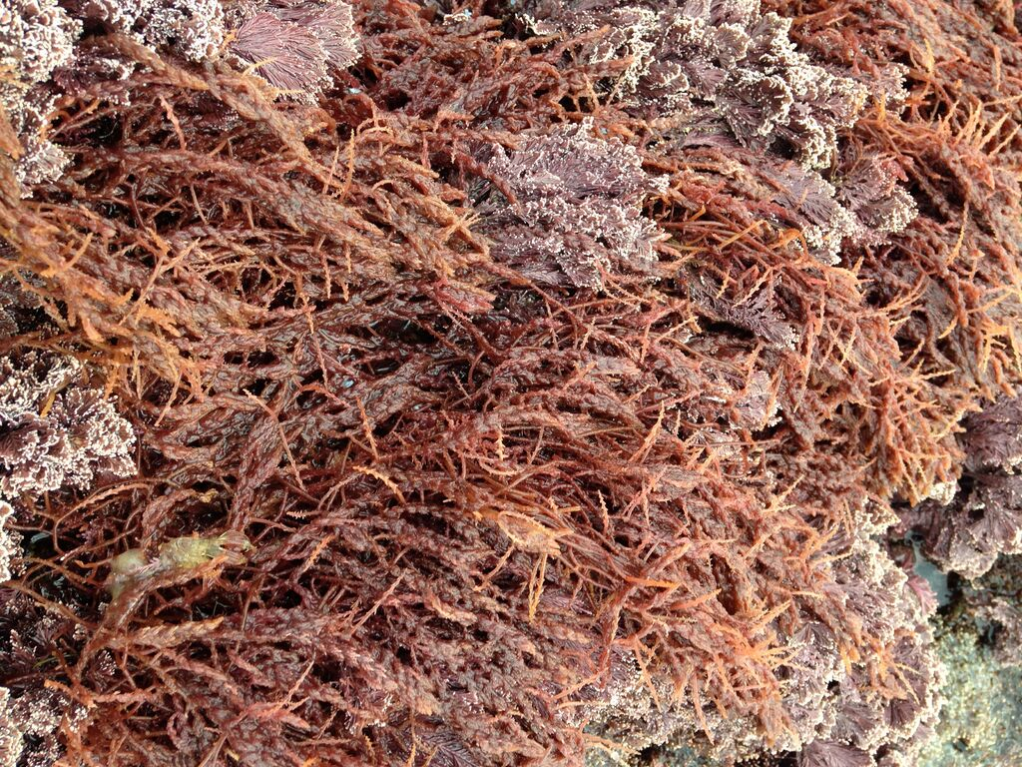
Red macrophytes *Asparagopsis armata* and *Ellisolandia elongata* at lower intertidal level (by the Island Aquatic Ecology Subgroup of cE3c-ABG).

**Figure 5.**
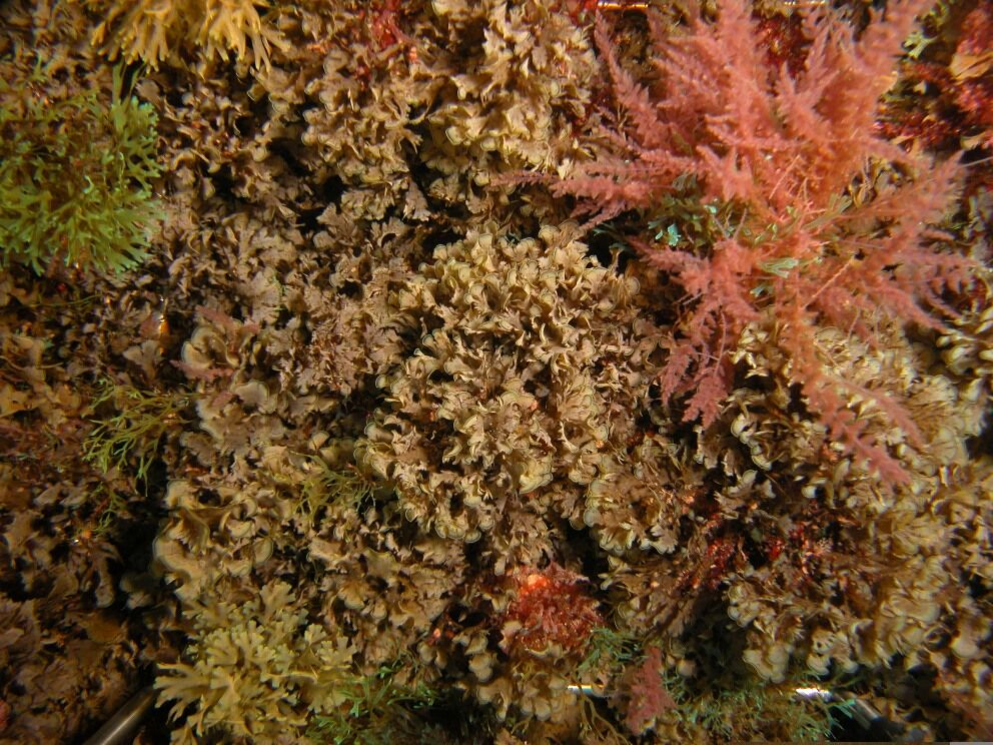
Frondose brown macrophytes (*Zonaria tournefortii* and *Dictyota* spp.) together with *Asparagopsis armata* at subtidal level (by the Island Aquatic Ecology Subgroup of cE3c-ABG).

As on the remaining Azores Islands, the intertidal and shallow subtidal rocky-shore communities of Graciosa are dominated by macroalgae (Neto et al. 2005). The high intertidal level communities are characterised by a patchy mosaic of algae (principally *Fucus spiralis* Linnaeus, *Gelidium microdon* Kützing and *Gymnogongrus* spp.) and a few animals (mainly chthamalid barnacles) (Fig. 2). Lower, the shore is covered by algal turfs (growth forms of either diminutive algae or diminutive forms of larger species that create a dense, compact mat 20-30 mm thick), either monospecific or composed of several species, for example, calcareous algae (e.g. *Ellisolandia* and *Jania*) or by soft algae (e.g. *Centroceras clavulatum* (C.Agardh) Montagne, *Chondracanthus* and *Laurencia*) (Fig. 3). At this level, a few limpets may be seen. The erect, corticated macrophytes *Ellisolandia elongata* (J.Ellis & Solander) K.R.Hind & G.W.Saunders, *Pterocladiella capillacea* (S.G.Gmelin) Santelices & Hommersand (Fig. 4) and *Treptacantha abies-marina* (S.G.Gmelin) Kützing are common in the transition zone to the subtidal, which is usually dominated by large foliose species (Neto et al. 2009). Subtidally, algal communities are characterised by associations of two or three frondose macrophytes (Fig. 5), predominantly the brown seaweeds, for example, *Dictyota* spp., *Halopteris* spp. and *Zonaria tournefortii* (J.V.Lamouroux) Montagne (Neto et al. 2009).

### Design description

The algae, referred to in this paper, were collected during field studies at littoral and sublittoral levels down to approximately 40 m on the Island of Graciosa. Each sampling location was visited several times. On each occasion, a careful and extensive survey was undertaken to provide good coverage of the area. Both presence recording and physical collections were made by walking over the shores or by scuba diving. The specimens collected were taken to the laboratory for identification and preservation and the resulting vouchers were deposited at the AZB Herbarium Ruy Telles Palhinha and the Molecular Systematics Laboratory at the Faculty of Sciences and Technology of the University of the Azores.

### Funding

This study was mainly financed by the following projects/scientific expeditions:

- Expedition GRACIOSA/2004, Departamento de Biologia da Universidade dos Açores Ilha do Pico, Açores, June 2004;
- Campaign PADEL/2006, under the project “PADEL: Património natural e desenvolvimento sustentável do litoral dos Açores: a Ilha Graciosa como caso de estudo”. 2006 - 2007. The Azores Regional Government;
- Campaign MACROBIOMOL/2014, under the project “MACROBIOMOL, Macroalgal biodiversity under molecular lens - towards a better understanding of North Atlantic biogeography” (PTDC/MAR/114 613/2009). 2011 - 2015. Operational Programme COMPETE (ERDF funds), FCT (UID/BIA/50027/2013) and POCI-01-0145-FEDER-006821;
- Campaign PIMA-BALA/2017, under the projects “PIMA (3/DRAM/2015), Elaboração do programa de implementação da Diretiva-Quadro Estratégia Marinha - Programa invasoras marinhas nos Açores” and “BALA (2/DRAM/2015), Elaboração do programa de implementação da diretiva-quadro estratégia marinha - biodiversidade dos ambientes litorais dos Açores”. ERDF funds, and the Azores Regional Government;
- Project “ACORES-01-0145-FEDER-000072 - AZORES BIOPORTAL – PORBIOTA. Operational Programme Azores 2020 (85% ERDF and 15% regional funds);
- Portuguese National Funds, through FCT –Fundação para a Ciência e a Tecnologia, within the projects UID/BIA/00329/2013, 2015- 2019, UID/BIA/00329/2020-2023 and UID/BIA/50027/2019 and POCI-01-0145-FEDER-006821;
- Portuguese Regional Funds, through DRCT – Direção Regional da Ciência e Tecnologia, within several projects, 2019 and 2020;
- CIRN/DB/UAc (Research Centre for Natural Resources, Universidade dos Açores, Departamento de Biologia);
- CIIMAR (Interdisciplinary Centre of Marine and Environmental Research, Porto, Portugal).

## Sampling methods

### Study extent

**Figure 6.**
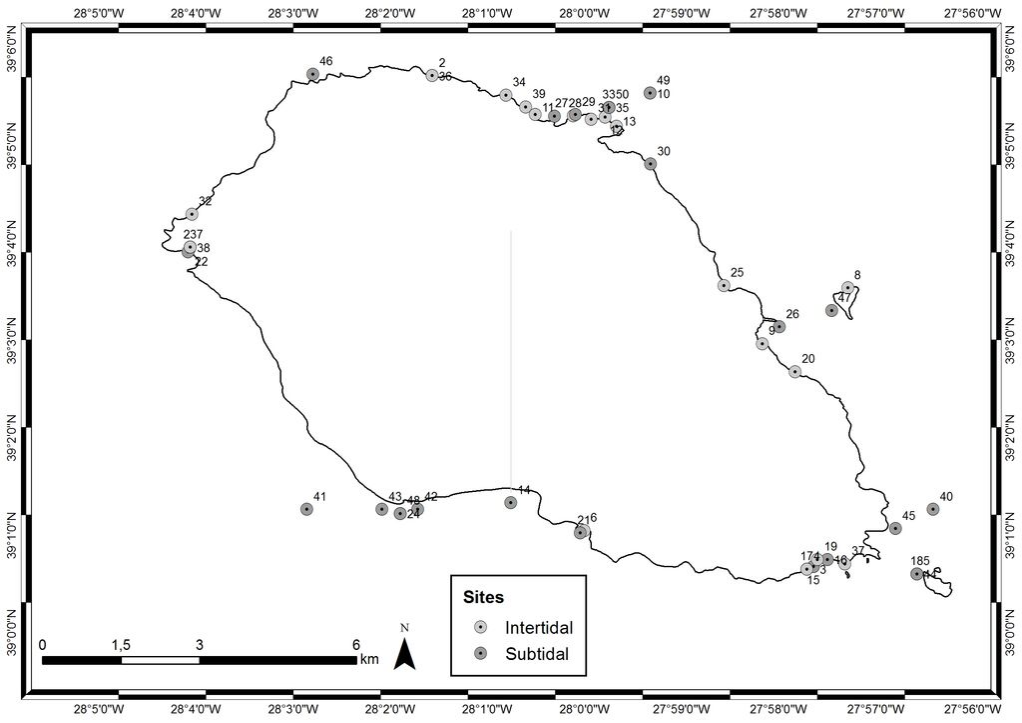
Sampling locations around Graciosa Island (by Nuno V. Álvaro).

This study covers a relatively-large area, approximately 19 km^2^, encompassing littoral and sublittoral levels down to approximately 40 m around the Island of Graciosa (Table 2, Fig. 6).

### Sampling description

**Figure 7.**
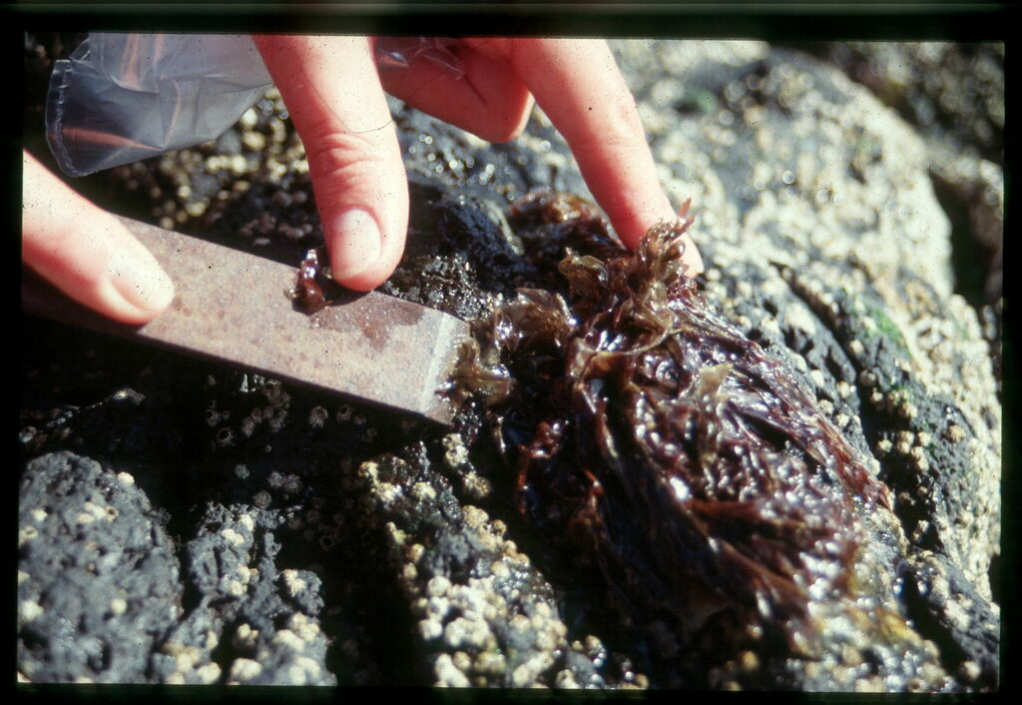
Collecting macroalgae at the rocky intertidal (by the Island Aquatic Ecology Subgroup of cE3c-ABG) .

**Figure 8.**
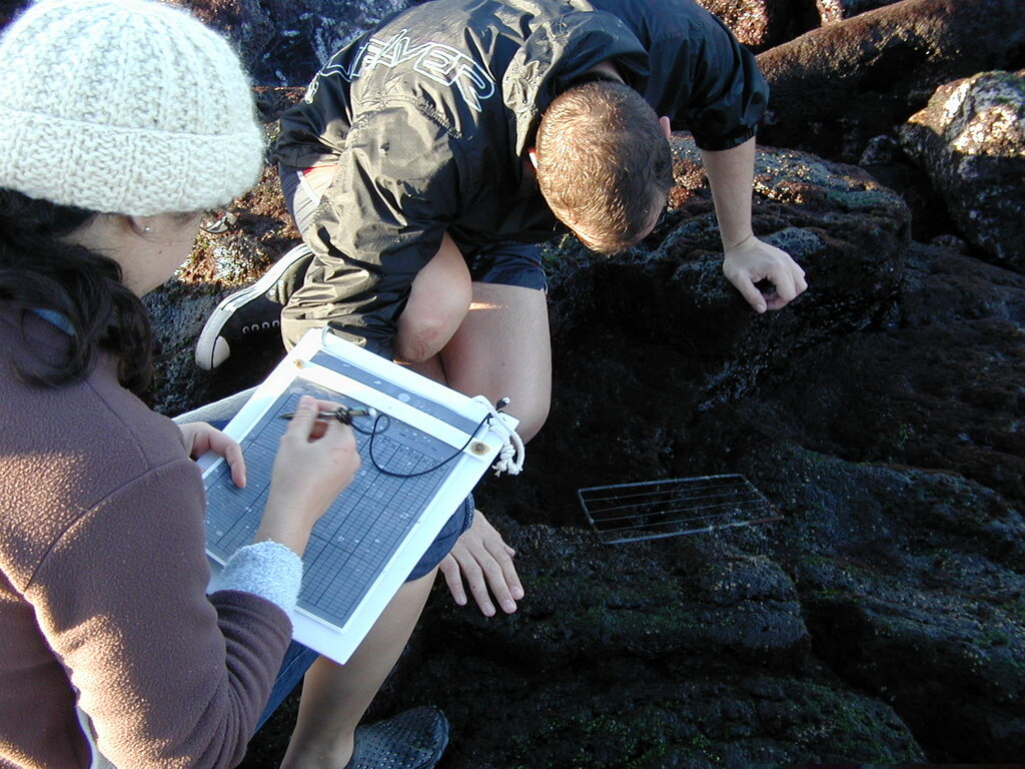
Quantitative recording of the presence and coverage of macroalgal species at the intertidal rocky habitat (by the Island Aquatic Ecology Subgroup of cE3c-ABG).

Intertidal collections were made during low tide by walking over the shores. Subtidal collections were made by scuba diving around the area. Sampling involved specimen collecting and species-presence recording. For the former, at each location, samples were obtained by scraping (Fig. 7) one or two specimens of all different species found into labelled bags. Species-recording data were gathered by registering all species present in the sampled locations visited (Fig. 8). Complementary data, for example, shore level (high, mid, low), orientation and type of substrate (bedrock, boulders, cobbles, mixed), habitat (tide pool, open rock, gully, crevice, cave) were also recorded.

### Quality control

Each sampled taxon was identified by trained taxonomists and involved morphological and anatomical observations of whole specimens by eye or of slide preparations under the microscope for the diagnostic features described in literature.

### Step description

Specimens were brought back to the laboratory, sorted and studied following standard procedures used in macroalgae identification.

Species identification was based on morphological and anatomical characters and reproductive structures. For small and simple thalli, this required the observation of the entire thallus with the naked eye and/or using dissecting and compound microscopes. For larger and more complex algae, investigation of the thallus anatomy required histological preparations (longitudinal and transverse sections) for the observation of cells, reproductive structures and other diagnostic characters.

As the Azorean algal flora has representatives from several geographical regions, often causing difficulty in identification, floras and keys for the North Atlantic, Tropical Atlantic and Western Mediterranean were used (e.g. Schmidt 1931, Taylor 1967, Taylor 1978, Levring 1974, Dixon and Irvine 1977, Lawson and John 1982, Irvine 1983, Gayral and Cosson 1986, Fletcher 1987, Afonso-Carrillo and Sansón 1989, Burrows 1991, Boudouresque et al. 1992, Cabioc'h et al. 1992, Maggs and Hommersand 1993, Irvine and Chamberlain 1994, Brodie et al. 2007, Lloréns et al. 2012 and Rodríguez-Prieto et al. 2013).

For more critical and taxonomically-difficult taxa, specimens were taken to the Natural History Museum (London) for comparison with collections there.

A reference collection was made for all collected specimens by assigning them a herbarium code number and depositing them at the AZB Herbarium Ruy Telles Palhinha and the Molecular Systematics Laboratory, University of Azores. Depending on the species and on planned further research, different types of collections were made, namely (i) liquid collections using 5% buffered formaldehyde seawater and then replacing it by the fixing agent Kew (Bridsen and Forman 1999); (ii) dried collections, either by pressing the algae (most species) as described by Gayral and Cosson (1986) or by letting them air-dry (calcareous species); and (iii) silica gel collections for molecular study.

Nomenclatural and taxonomic status used here follow *Algaebase* (Guiry and Guiry 2020). The database was organised on FileMaker Pro.

## Geographic coverage

### Description

Graciosa Island, Azores, Macaronesia, Portugal (approximately 39°0′38″N, 27°59′1″W).

### Coordinates

39.002 and 39.104 Latitude; -28.076 and -27.927 Longitude.

## Taxonomic coverage

### Description

All macroalgae were identified to genus or species level. In total, 250 taxa were identified belonging to 31 orders and 66 families, distributed by the phyla Rhodophyta (17 orders and 41 families), Chlorophyta (4 orders and 9 families) and Ochrophyta (10 orders and 16 families).

### Taxa included

**Table:** 

Rank	Scientific Name	Common Name
phylum	Rhodophyta	Red algae
phylum	Chlorophyta	Green algae
phylum	Ochrophyta	Brown algae

## Temporal coverage

**Living time period:** 2004 - 2017.

### Notes

The sampling was performed on several occasions in the period between 2004 and 2017.

## Collection data

### Collection name

AZB | Marine macroalgae collection of Graciosa Island (Azores) – Expedition GRACIOSA/2004; AZB | Marine macroalgae collection of Graciosa Island (Azores) – Project PADEL; AZB | Marine macroalgae collection of Graciosa Island (Azores) – Occasional sampling; Marine macroalgae collection of Graciosa Island (Azores)-Project MACROBIOMOL; Marine macroalgae collection of Graciosa Island (Azores)-Campaign PIMA/BALA; Marine macroalgae occurrence in Graciosa Island (Azores) – Project PADEL

### Collection identifier

5ee0202d-c659-436f-9b78-664df8e2791d; 915baa3f-e5b0-4673-ba80-00c05420e1ef; c1904e12-0389-4e52-b78d-8cd18942fd3d; dc0e952e-51be-4677-8789-a02e57869e7a; fc35e5ae-2143-4b62-87af-ede8db82fc2c; e29a0327-dcd3-4626-831a-4606c7862220

### Parent collection identifier

AZB Herbarium Ruy Telles Palhinha, Faculty of Sciences and Technology of the University of the Azores; AZB Herbarium Ruy Telles Palhinha, Faculty of Sciences and Technology of the University of the Azores; AZB Herbarium Ruy Telles Palhinha, Faculty of Sciences and Technology of the University of the Azores; MACROBIOMOL Macroalgae collection, Faculty of Sciences and Technology of the University of the Azores; PIMA/BALA Macroalgae collection, Faculty of Sciences and Technology of the University of the Azores; AZB Herbarium Ruy Telles Palhinha, Faculty of Sciences and Technology of the University of the Azores.

### Specimen preservation method

Air-dry, Dried and pressed; Liquid (Formalin; fixing agent Kew), Silica.

### Curatorial unit

AZB Herbarium Ruy Telles Palhinha, Faculty of Sciences and Technology of the University of the Azores.

## Usage rights

### Use license

Creative Commons Public Domain Waiver (CC-Zero)

## Data resources

### Data package title

Marine algal (seaweed) flora of Graciosa Island, Azores

### Resource link

http://ipt.gbif.pt/ipt/resource?r=graciosa_seaweed_flora&v=1.9

### Alternative identifiers

http://ipt.gbif.pt/ipt/resource?r=graciosa_seaweed_flora; https://doi.org/10.15468/uxjpmx

### Number of data sets

1

### Data set 1.

#### Data set name

Marine algal (seaweed) flora of Graciosa Island, Azores

#### Data format

Darwin Core Archive

#### Number of columns

49

#### Download URL

http://ipt.gbif.pt/ipt/resource?r=graciosa_seaweed_flora&v=1.9

#### Data format version

1.6

#### Description

This data paper presents both physical and occurrence data from macroalgal surveys undertaken on Graciosa Island between 2004 and 2017 (Neto et al. 2020c. The dataset submitted to GBIF is structured as a sample event dataset, with two tables: event (as core) and occurrences. The data in this sampling event resource have been published as a Darwin Core Archive (DwCA), which is a standardised format for sharing biodiversity data as a set of one or more data tables. The core data table contains 50 records (eventID). The extension data table has 1692 occurrences. An extension record supplies extra information about a core record. The number of records in each extension data table is illustrated in the IPT link. This IPT archives the data and thus serves as the data repository. The data and resource metadata are available for downloading in the downloads section.

**Data set 1.** 

Column label	Column description
Table of Sampling Events	Table with sampling events data (beginning of table)
eventID	Identifier of the event, unique for the dataset
country	Country of the sampling site
countryCode	Code of the country where the event occurred
stateProvince	Name of the region
island	Name of the island
municipality	Name of the municipality
locality	Name of the locality
locationID	Identifier of the location
decimalLatitude	The geographic latitude of the sampling site
decimalLongitude	The geographic longitude of the sampling site
geodeticDatum	The spatial reference system upon which the geographic coordinates are based
coordinateUncertaintyInMetres	The horizontal distance (in metres) from the given decimalLatitude and decimalLongitude describing the smallest circle containing the whole of the Location
eventDate	Time interval when the event occurred
year	The year of the event
samplingProtocol	Sampling method used during an event
locationRemarks	Zonation level
minimumDepthInMetres	The minimum depth in metres where the specimen was found
maximumDepthInMetres	The maximum depth in metres where the specimen was found
eventRemarks	Notes about the event
Table of Species Occurrence	Table with species occurrence data (beginning of new table)
occurrenceID	Identifier of the record, coded as a global unique identifier
institutionID	The identifier for the institution having custody of the object or information referred to in the record
institutionCode	The acronym of the institution having custody of the object or information referred to in the record
collectionID	An identifier of the collection to which the record belongs
collectionCode	The name of the collection from which the record was derived
datasetName	The name identifying the dataset from which the record was derived
eventID	Identifier of the event, unique for the dataset
kingdom	Kingdom name
phyllum	Phylum name
class	Class name
order	Order name
family	Family name
genus	Genus name
specificEpithet	The name of the first or species epithet of the scientificName
infraspecificEpithet	The name of the lowest or terminal infraspecific epithet of the scientificName, excluding any rank designation
acceptedNameUsage	The specimen accepted name, with authorship
previousIdentifications	Previous name of the specimen, with authorship
scientificName	The name without authorship applied on the first identification of the specimen
basisOfRecord	The specific nature of the data record
habitat	Description of the habitat where the specimen was found
recordedBy	Person(s) responsible for sampling
catalogNumber	Identifying code for a unique sample lot in a biological collection
identifiedBy	Person(s) responsible for taxa identification
type	The nature of the resource
preparations	The preservation method used for the specimen
establishmentMeans	The establishment status of the organism in the study region
occurrenceRemarks	New record status assignment
licence	Reference to the licence under which the record is published

## Additional information

This paper accommodates the 1692 specimens of macroalgae recorded from Graciosa Island in 250 taxa with 195 confirmed species and 55 taxa identified only to genus level. The confirmed species (Tables 3, 4) comprise 126 Rhodophyta, 31 Chlorophyta and 38 Ochrophyta (Phaeophyceae). Of these, 79 species are newly recorded to the algal flora of the Island (47 Rhodophyta, 10 Chlorophyta and 22 Ochrophyta). Most species are native, including the Azorean endemic Predaea feldmannii subsp. azorica Gabriel and the Macaronesian endemics *Botryocladia macaronesica* Afonso-Carrillo, Sobrino, Tittley & Neto, *Phyllophora gelidioides* P.Crouan & H.Crouan ex Karsakoff, *Laurencia viridis* Gil-Rodríguez & Haroun and *Codium elisabethiae* O.C. Schmidt. Fourteen species are introductions to the algal flora and 20 have an uncertain status.

Many species were only sporadically recorded around the Island, but 12 were commonly found, namely: the Rhodophyta *Ceramium virgatum* Roth, *Chondria dasyphylla* (Woodward) C. Agardh, *Hypnea musciformis* (Wulfen) J. V. Lamouroux, *Pterocladiella capillacea* (S. G. Gmelin) Santelices & Hommersand and *Scinaia interrupta* (A. P. de Candolle) M. J. Wynne; the Chlorophyta *Chaetomorpha pachynema* (Montagne) Kützing, *Codium adhaerens* C. Agradh and *Ulva rigida* C. Agardh; and the Ochrophyta *Colpomenia sinuosa* (Mertens ex Roth) Derbès & Solier in Castagne, *Halopteris scoparia* (Linnaeus) Sauvageau, *Treptacantha abies-marina* (S. G. Gmelin) Kützing and *Zonaria tournefortii* (J. V. Lamouroux) Montagne.

A mismatch regarding the GBIF backbone taxonomy of some of the macroalgae species names was identified as detailed in Suppl. material 1.

## Supplementary Material

43667BDE-9FB2-5384-B7E5-B00CDA5C0384 10.3897/BDJ.8.e57201.suppl1 Supplementary material 1 DP-GRA-id_14140_normalized.csv Data type Macroalgae taxonomic mismatching Brief description GBIF does not have the more actualised nomenclature for some of the macroalgae species names. Therefore, the matching tools of its platform were applied to the species list, as required by Pensoft's data auditor, to identify the problematic taxonomic situations. The resulting file (DP-GRA-id_14140_normalized.csv) is included here, since the names will not be immediately updated in the GBIF Taxonomic Backbone. A request was already sent to GBIF helpdesk to solve this situation. File: oo_437509.csv https://binary.pensoft.net/file/437509 Ana I Neto

## Figures and Tables

**Table 1.** Number of macroalgal species on the Azorean Islands (Neto et al. 2020b, c and authors' unpublished data).

Phyllum	Santa Maria	São Miguel	Terceira	Graciosa	São Jorge	Pico	Faial	Flores	Corvo
Rhodophyta	68	168	73	79	35	142	59	59	13
Chlorophyta	20	39	24	21	17	41	16	16	2
Ochrophyta	28	53	16	16	10	42	8	16	4
Total	116	260	113	116	62	225	83	91	19

**Table 2.** Location of the sampling sites on Graciosa Island.

Location N0	Location ID	Municipality	Locality	Latitude / Longitude	geodeticDatum	Littoral zone
1	GRA_SC_BPL	Santa Cruz	Baixa do Pesqueiro Longo	39,123197, -28,045288	WGS84	Intertidal
2	GRA_SC_BV	Santa Cruz	Barro Vermelho	39,095344, -28,026032	WGS84	Intertidal
3	GRA_SC_Cb1	Santa Cruz	Carapacho|Baía 1	39,011211, -27,960628	WGS84	Subtidal
4	GRA_SC_Cem	Santa Cruz	Carapacho|Entre-marés	39,010819, -27,961696	WGS84	Intertidal
5	GRA_SC_Cib	Santa Cruz	Carapacho|Ilhéu de Baixo	39,009987, -27,942812	WGS84	Subtidal
6	GRA_SC_Fop	Santa Cruz	Folga|porto	39,017394, -27,999973	WGS84	Intertidal
7	GRA_SC_PAp	Santa Cruz	Porto Afonso|porto	39,065946, -28,06759	WGS84	Intertidal
8	GRA_SC_Pi	Santa Cruz	Praia|Ilhéu	39,058972, -27,954659	WGS84	Intertidal
9	GRA_SC_Psm	Santa Cruz	Praia|São Mateus	39,049412, -27,969379	WGS84	Intertidal
10	GRA_SC_SCbf	Santa Cruz	Santa Cruz|Baixa do Ferreiro	39,092344, -27,988639	WGS84	Subtidal
11	GRA_SC_SCp	Santa Cruz	Santa Cruz|porto	39,088680, -28,008354	WGS84	Intertidal
12	GRA_SC_SCpf	Santa Cruz	Santa Cruz|Ponta do Ferreiro	39,088263, -27,996325	WGS84	Intertidal
13	GRA_SC_SCpp	Santa Cruz	Santa Cruz|Ponta da Pesqueira	39,086693, -27,994449	WGS84	Intertidal
14	GRA_SC_BF	Santa Cruz	Baía do Filipe	39,022164, -28,012561	WGS84	Subtidal
15	GRA_SC_Cb1	Santa Cruz	Carapacho|Baía 1	39,011211, -27,960628	WGS84	Subtidal
16	GRA_SC_Cb2	Santa Cruz	Carapacho|Baía 2	39,012446, -27,958205	WGS84	Subtidal
17	GRA_SC_Cem	Santa Cruz	Carapacho|Entre-marés	39,010819, -27,961696	WGS84	Intertidal
18	GRA_SC_Cib	Santa Cruz	Carapacho|Ilhéu de Baixo	39,009987, -27,942812	WGS84	Subtidal
19	GRA_SC_Ct	Santa Cruz	Carapacho|Termas	39,01245, -27,959901	WGS84	Intertidal
20	GRA_SC_F	Santa Cruz	Fenais	39,044619, -27,963737	WGS84	Intertidal
21	GRA_SC_Fob	Santa Cruz	Folga|Baía	39,016995, -28,000629	WGS84	Subtidal
22	GRA_SC_PAbc	Santa Cruz	Porto Afonso|Baía das Caldeirinhas	39,065128, -28,067997	WGS84	Subtidal
23	GRA_SC_PAp	Santa Cruz	Porto Afonso|porto	39,065946, -28,06759	WGS84	Intertidal
24	GRA_SC_PB	Santa Cruz	Ponta Branca	39,020325, -28,031507	WGS84	Subtidal
25	GRA_SC_Pbl	Santa Cruz	Praia|Baía da Lagoa	39,059392, -27,975943	WGS84	Intertidal
26	GRA_SC_Pp	Santa Cruz	Praia|porto	39,052321, -27,966435	WGS84	Subtidal
27	GRA_SC_SCb	Santa Cruz	Santa Cruz|Barra	39,088431, -28,005052	WGS84	Subtidal
28	GRA_SC_SCba	Santa Cruz	Santa Cruz|Barra-Anel	39,088456, -28,001856	WGS84	Intertidal
29	GRA_SC_SCba	Santa Cruz	Santa Cruz|Barra-Anel	39,088694, -28,001464	WGS84	Subtidal
30	GRA_SC_SCbf	Santa Cruz	Santa Cruz|Baía da Fonte	39,080207, -27,98857	WGS84	Subtidal
31	GRA_SC_SCbsc	Santa Cruz	Santa Cruz|Baía de Santa Catarina	39,087896, -27,998773	WGS84	Intertidal
32	GRA_SC_SCbsv	Santa Cruz	Santa Cruz|Baía Senhora da Vitória	39,071636, -28,067268	WGS84	Intertidal
33	GRA_SC_SCpb	Santa Cruz	Santa Cruz|Ponta da Barca	39,089915, -27,995677	WGS84	Subtidal
34	GRA_SC_SCt	Santa Cruz	Santa Cruz|Terreiros	39,092024, -28,013401	WGS84	Intertidal
35	GRA_SC_SCpb	Santa Cruz	Santa Cruz|Ponta da Barca	39,089915, -27,995677	WGS84	Intertidal
36	GRA_SC_BV	Santa Cruz	Barro Vermelho	39,095344, -28,026032	WGS84	Intertidal
37	GRA_SC_Cp	Santa Cruz	Carapacho|Ponta	39,011703, -27,955194	WGS84	Intertidal
38	GRA_SC_PAp	Santa Cruz	Porto Afonso|porto	39,065946, -28,06759	WGS84	Intertidal
39	GRA_SC_SCap	Santa Cruz	Santa Cruz|Atrás do porto	39,09, -28,01	WGS84	Intertidal
40	GRA_SC_BB	Santa Cruz	Baixa do Badejo	39,021049, -27,94005	WGS84	Subtidal
41	GRA_SC_BPLpf	Santa Cruz	Baixa do Pesqueiro Longo|Picos de Fora	39,02105, -28,047544	WGS84	Subtidal
42	GRA_SC_BV	Santa Cruz	Barro Vermelho	39,02105, -28,0285	WGS84	Subtidal
43	GRA_SC_BVbpcn	Santa Cruz	Barro Vermelho|Baixa do Pintado|Costa Norte	39,02105, -28,03465	WGS84	Subtidal
44	GRA_SC_Cib	Santa Cruz	Carapacho|Ilhéu de Baixo	39,009987, -27,942812	WGS84	Subtidal
45	GRA_SC_Cpr	Santa Cruz	Carapacho|Ponta da Restinga	39,017723, -27,946526	WGS84	Subtidal
46	GRA_SC_IB	Santa Cruz	Ilhéu da Baleia	39,095601, -28,046544	WGS84	Subtidal
47	GRA_SC_IP	Santa Cruz	Ilhéu da Praia	39,055066, -27,957481	WGS84	Subtidal
48	GRA_SC_PB	Santa Cruz	Ponta Branca	39,020325, -28,031507	WGS84	Subtidal
49	GRA_SC_SCbf	Santa Cruz	Santa Cruz|Baixa do Ferreiro	39,092344, -27,988639	WGS84	Subtidal
50	GRA_SC_SCpb	Santa Cruz	Santa Cruz|Ponta da Barca	39,089915, -27,995677	WGS84	Subtidal

**Table 3.** Macroalgae species from Graciosa Island, with information on their relative abundance, origin and status.

**Phylum**	**Species (Accepted Name)**	**Number of records**	**Establishment Means**	**OccurrenceRemarks**
Chlorophyta	*Anadyomene saldanhae* A.B.Joly & E.C.Oliveira	1	Native	New record
Chlorophyta	*Bryopsis cupressina* J.V.Lamouroux	2	Native	
Chlorophyta	*Bryopsis hypnoides* J.V.Lamouroux	9	Native	
Chlorophyta	*Bryopsis plumosa* (Hudson) C.Agardh	9	Native	
Chlorophyta	*Chaetomorpha linum* (O.F.Müller) Kützing	1	Native	
Chlorophyta	*Chaetomorpha mediterranea* (Kützing) Kützing	1	Native	New record
Chlorophyta	*Chaetomorpha pachynema* (Montagne) Kützing	27	Native	
Chlorophyta	*Cladophora albida* (Nees) Kutzing	5	Native	
Chlorophyta	*Cladophora coelothrix* Kützing	1	Native	
Chlorophyta	*Cladophora conferta* P.Crouan & H.Crouan	4	Native	
Chlorophyta	*Cladophora laetevirens* (Dillwyn) Kützing	1	Uncertain	
Chlorophyta	*Cladophora liebetruthii* Grunow	8	Native	
Chlorophyta	*Cladophora prolifera* (Roth) Kützing	15	Native	
Chlorophyta	*Codium adhaerens* C. Agradh	24	Native	
Chlorophyta	*Codium decorticatum* (Woodward) M.Howe	1	Native	New record
Chlorophyta	*Codium elisabethiae* O.C.Schmidt	2	Macaronesian endemism	New record
Chlorophyta	Codium fragile subsp. fragile (Suringar) Hariot	1	Introduced	New record
Chlorophyta	*Codium taylorii* P.C. Silva	1	Native	New record
Chlorophyta	*Codium vermilara* (Olivi) Delle Chiaje	11	Native	New record
Chlorophyta	*Derbesia tenuissima* (Moris & De Notaris) P.Crouan & H.Crouan	5	Uncertain	
Chlorophyta	*Lychaete pellucida* (Hudson) M.J.Wynne	5	Native	
Chlorophyta	*Microdictyon umbilicatum* (Velley) Zanardini	11	Native	New record
Chlorophyta	*Phyllodictyon anastomosans* (Harvey) Kraft & M.J.Wynne	5	Native	New record
Chlorophyta	*Ulva clathrata* (Roth) C.Agardh	10	Native	
Chlorophyta	*Ulva compressa* Linnaeus	19	Native	
Chlorophyta	*Ulva intestinalis* Linnaeus	4	Native	
Chlorophyta	*Ulva linza* Linnaeus	1	Native	New record
Chlorophyta	*Ulva prolifera* O.F.Müller	1	Native	
Chlorophyta	*Ulva rigida* C.Agardh	37	Native	
Chlorophyta	*Valonia macrophysa* Kützing	3	Native	
Chlorophyta	*Valonia utricularis* (Roth) C.Agardh	2	Native	
Ochrophyta	*Ascophyllum nodosum* (Linnaeus) Le Jolis	2	Native	
Ochrophyta	*Bachelotia antillarum* (Grunow) Gerloff	5	Native	
Ochrophyta	*Carpomitra costata* (Stackhouse) Batters	6	Native	New record
Ochrophyta	*Cladostephus spongiosum* (Hudson) C.Agardh	15	Native	New record
Ochrophyta	*Colpomenia sinuosa* (Mertens ex Roth) Derbès & Solier	39	Native	New record
Ochrophyta	*Cutleria multifida* (Turner) Greville	1	Uncertain	
Ochrophyta	*Cystoseira compressa* (Esper) Gerloff & Nizamuddin	12	Native	New record
Ochrophyta	*Cystoseira humilis* Schousboe ex Kützing	8	Native	New record
Ochrophyta	*Dictyopteris polypodioides* (A.P.de Candolle) J.V.Lamouroux	10	Native	New record
Ochrophyta	*Dictyota bartayresiana* J.V.Lamouroux	1	Native	New record
Ochrophyta	*Dictyota dichotoma* (Hudson) J.V.Lamouroux	11	Native	
Ochrophyta	Dictyota dichotoma var. intricata (C.Agardh) Greville	1	Native	New record
Ochrophyta	*Dictyota fasciola* (Roth) J.V.Lamouroux	2	Native	New record
Ochrophyta	*Ectocarpus siliculosus* (Dillwyn) Lyngbye	1	Uncertain	New record
Ochrophyta	*Feldmannia irregularis* (Kützing) Hamel	4	Native	New record
Ochrophyta	*Feldmannia paradoxa* (Montagne) Hamel	4	Native	New record
Ochrophyta	*Fucus spiralis* Linnaeus	15	Uncertain	
Ochrophyta	*Halopteris filicina* (Grateloup) Kützing	15	Native	
Ochrophyta	*Halopteris scoparia* (Linnaeus) Sauvageau	24	Native	
Ochrophyta	*Hydroclathrus clathratus* (C.Agardh) M.Howe in N.L.Britton & C.F.Millspaugh	2	Native	New record
Ochrophyta	*Hydroclathrus tilesii* (Endlicher) Santiañez & Wynne	9	Introduced	New record
Ochrophyta	*Laminaria ochroleuca* Bachelot de la Pylaie	9	Native	New record
Ochrophyta	*Leathesia marina* (Lyngbye) Decaisne	4	Uncertain	
Ochrophyta	*Lobophora variegata* (J.V.Lamouroux) Womersley ex E.C.Oliveira	3	Native	New record
Ochrophyta	*Myrionema strangulans* Greville	3	Native	New record
Ochrophyta	*Nemoderma tingitanum* Schousboe ex Bornet	4	Native	
Ochrophyta	*Padina pavonica* (Linnaeus) Thivy	14	Native	
Ochrophyta	*Papenfussiella kuromo* (Yendo) Inagaki	16	Introduced	
Ochrophyta	*Petalonia binghamiae* (J.Agardh) K.L.Vinogradova	14	Introduced	
Ochrophyta	*Ralfsia verrucosa* (Areschoug) Areschoug	1	Native	New record
Ochrophyta	*Sargassum cymosum* C.Agardh	5	Native	
Ochrophyta	*Sargassum desfontainesii* (Turner) C.Agardh	1	Native	New record
Ochrophyta	*Sargassum furcatum* Kützing	10	Native	New record
Ochrophyta	*Sphacelaria cirrosa* (Roth) C.Agardh	5	Native	
Ochrophyta	*Sphaerotrichia divaricata* (C.Agardh) Kylin	3	Uncertain	New record
Ochrophyta	*Taonia atomaria* (Woodward) J.Agardh	4	Native	New record
Ochrophyta	*Treptacantha abies-marina* (S.G.Gmelin) Kützing	25	Native	
Ochrophyta	*Zonaria tournefortii* (J.V.Lamouroux) Montagne	27	Native	
Rhodophyta	*Acrosorium ciliolatum* (Harvey) Kylin	11	Native	
Rhodophyta	*Aglaothamnion bipinnatum* (P.Crouan & H.Crouan) Feldmann & G.Feldmann	3	Native	New record
Rhodophyta	*Aglaothamnion cordatum* (Børgesen) Feldmann-Mazoyer	5	Introduced	
Rhodophyta	*Aglaothamnion tenuissimum* (Bonnemaison) Feldmann-Mazoyer	5	Uncertain	
Rhodophyta	*Ahnfeltiopsis devoniensis* (Greville) P.C.Silva & DeCew	6	Native	
Rhodophyta	*Amphiroa beauvoisii* J.V.Lamouroux	1	Native	
Rhodophyta	*Amphiroa cryptarthrodia* Zanardini	2	Native	
Rhodophyta	*Amphiroa fragilissima* (Linnaeus) J.V.Lamouroux	2	Native	New record
Rhodophyta	*Amphiroa rigida* J.V.Lamouroux	3	Native	New record
Rhodophyta	*Anotrichium furcellatum* (J.Agardh) Baldock	1	Uncertain	
Rhodophyta	*Antithamnion diminuatum* Wollaston	7	Introduced	
Rhodophyta	*Antithamnionella spirographidis* (Schiffner) E.M.Wollaston	1	Introduced	
Rhodophyta	*Asparagopsis armata* Harvey	14	Introduced	
Rhodophyta	*Asparagopsis armata* Harvey, phase *Falkenbergia rufolanosa* (Harvey) F.Schmitz	2	Introduced	
Rhodophyta	*Asparagopsis taxiformis* (Delile) Trevisan	6	Native	
Rhodophyta	*Asteromenia peltata* (W.R.Taylor) Huisman & A.J.K.Millar	5	Native	New record
Rhodophyta	*Bornetia secundiflora* (J.Agardh) Thuret	1	Native	
Rhodophyta	*Botryocladia macaronesica* Afonso-Carrillo, Sobrino, Tittley & Neto	1	Macaronesian endemism	New record
Rhodophyta	*Callithamnion corymbosum* (Smith) Lyngbye	13	Native	
Rhodophyta	*Callithamnion granulatum* (Ducluzeau) C.Agardh	1	Native	New record
Rhodophyta	*Callithamnion tetragonum* (Withering) S.F.Gray	4	Native	New record
Rhodophyta	*Callithamnion tetricum* (Dillwyn) S.F.Gray	2	Native	New record
Rhodophyta	*Carradoriella denudata* (Dillwyn) A.M.Savoie & G.W.Saunders	8	Uncertain	
Rhodophyta	*Carradoriella elongata* (Hudson) A.M.Savoie & G.W.Saunders	17	Native	
Rhodophyta	*Catenella caespitosa* (Withering) L.M.Irvine	1	Native	
Rhodophyta	*Caulacanthus ustulatus* (Mertens ex Turner) Kützing	7	Uncertain	
Rhodophyta	*Centroceras clavulatum* (C.Agardh) Montagne	16	Native	
Rhodophyta	*Ceramium botryocarpum* A.W.Griffiths ex Harvey	1	Native	New record
Rhodophyta	*Ceramium ciliatum* (J.Ellis) Ducluzeau	10	Native	
Rhodophyta	*Ceramium codii* (H.Richards) Mazoyer	3	Native	New record
Rhodophyta	*Ceramium diaphanum* (Lightfoot) Roth	12	Native	
Rhodophyta	*Ceramium virgatum* Roth	31	Native	
Rhodophyta	*Champia parvula* (C.Agardh) Harvey	2	Native	New record
Rhodophyta	*Chondracanthus acicularis* (Roth) Fredericq	17	Native	
Rhodophyta	*Chondracanthus teedei* (Mertens ex Roth) Kützing	10	Native	New record
Rhodophyta	*Chondria coerulescens* (J.Agardh) Sauvageau	4	Uncertain	
Rhodophyta	*Chondria dasyphylla* (Woodward) C.Agardh	41	Uncertain	
Rhodophyta	*Corallina ferreyrae* E.Y.Dawson, Acleto & Foldvik	6	Native	New record
Rhodophyta	*Cryptonemia palmetta* (S.G.Gmelin) Woelkering, G.Furnari, Cormaci & J.McNeill	2	Native	New record
Rhodophyta	*Cryptopleura ramosa* (Hudson) L.Newton	11	Native	
Rhodophyta	*Dasya caraibica* Børgesen	2	Native	
Rhodophyta	*Dasya corymbifera* J.Agardh	4	Native	
Rhodophyta	*Dasya hutchinsiae* Harvey	4	Native	
Rhodophyta	*Dermocorynus dichotomus* (J.Agardh) Gargiulo, M.Morabito & Manghisi	21	Native	
Rhodophyta	*Ellisolandia elongata* (J.Ellis & Solander) K.R.Hind & G.W.Saunders	23	Native	
Rhodophyta	*Erythrocystis montagnei* (Derbès & Solier) P.C.Silva	7	Native	
Rhodophyta	*Gaillona hookeri* (Dillwyn) Athanasiadis	3	Native	New record
Rhodophyta	*Gastroclonium reflexum* (Chauvin) Kützing	1	Native	
Rhodophyta	*Gelidium arbuscula* Bory ex Børgesen	4	Native	New record
Rhodophyta	*Gelidium corneum* (Hudson) J.V.Lamouroux	3	Native	New record
Rhodophyta	*Gelidium microdon* Kützing	17	Native	
Rhodophyta	*Gelidium pusillum* (Stackhouse) Le Jolis	12	Native	
Rhodophyta	*Gelidium spinosum* (S.G.Gmelin) P.C.Silva	12	Native	
Rhodophyta	*Gigartina pistillata* (S.G.Gmelin) Stackhouse	3	Native	
Rhodophyta	*Grateloupia filicina* (J.V.Lamouroux) C.Agardh	10	Native	
Rhodophyta	*Griffithsia phyllamphora* J.Agardh	7	Native	New record
Rhodophyta	*Gymnogongrus crenulatus* (Turner) J.Agardh	8	Native	
Rhodophyta	*Gymnogongrus griffithsiae* (Turner) C.Martius	19	Native	
Rhodophyta	*Gymnothamnion elegans* (Schousboe ex C.Agardh) J.Agardh	1	Native	
Rhodophyta	*Halarachnion ligulatum* (Woodward) Kützing	1	Native	New record
Rhodophyta	*Halurus equisetifolius* (Lightfoot) Kützing	3	Native	New record
Rhodophyta	*Halurus flosculosus* (J.Ellis) Maggs & Hommersand	7	Native	
Rhodophyta	*Herposiphonia secunda* (C.Agardh) Ambronn	7	Native	
Rhodophyta	*Heterosiphonia crispella* (C.Agardh) M.J.Wynne	10	Native	
Rhodophyta	*Hildenbrandia crouaniorum* J.Agardh	3	Native	New record
Rhodophyta	*Hypnea arbuscula* P.J.L.Dangeard	2	Native	
Rhodophyta	*Hypnea musciformis* (Wulfen) J.V.Lamouroux	25	Uncertain	
Rhodophyta	*Hypnea spinella* (C.Agardh) Kützing	10	Native	New record
Rhodophyta	*Hypoglossum heterocystideum* (J.Agardh) J.Agardh	4	Introduced	New record
Rhodophyta	*Jania capillacea* Harvey	2	Native	
Rhodophyta	*Jania longifurca* Zanardini	11	Uncertain	
Rhodophyta	Jania pedunculata var. adhaerens (J.V.Lamouroux) A.S.Harvey, Woelkerling & Reviers	2	Native	New record
Rhodophyta	*Jania pumila* J.V.Lamouroux	2	Native	
Rhodophyta	*Jania rubens* (Linnaeus) J.V.Lamouroux	5	Native	
Rhodophyta	*Jania virgata* (Zanardini) Montagne	4	Uncertain	
Rhodophyta	*Kallymenia reniformis* (Turner) J.Agardh	2	Native	New record
Rhodophyta	*Laurencia dendroidea* J.Agardh	4	Introduced	
Rhodophyta	*Laurencia minuta* Vandermeulen, Garbary & Guiry	1	Introduced	
Rhodophyta	*Laurencia obtusa* (Hudson) J.V.Lamouroux	1	Native	
Rhodophyta	*Laurencia pyramidalis* Bory ex Kützing	12	Native	New record
Rhodophyta	*Laurencia tenera* C.K.Tseng	1	Native	New record
Rhodophyta	*Laurencia viridis* Gil-Rodríguez & Haroun	4	Macaronesian endemism	
Rhodophyta	*Leptosiphonia brodiei* (Dillwyn) A.M.Savoie & G.W.Saunders	1	Uncertain	
Rhodophyta	*Liagora distenta* (Mertens ex Roth) J.V.Lamouroux	7	Native	New record
Rhodophyta	*Liagora viscida* (Forsskål) C.Agardh	3	Native	New record
Rhodophyta	*Lithophyllum incrustans* Philippi	1	Native	New record
Rhodophyta	*Lomentaria articulata* (Hudson) Lyngbye	22	Native	
Rhodophyta	*Melanothamnus harveyi* (Bailey) Díaz-Tapia & Maggs	4	Introduced	
Rhodophyta	*Meredithia microphylla* (J.Agardh) J.Agardh	8	Native	New record
Rhodophyta	*Mesophyllum expansum* (Philippi) Cabioch & M.L.Mendoza	1	Native	
Rhodophyta	*Mesophyllum lichenoides* (J.Ellis) Me.Lemoine	3	Native	New record
Rhodophyta	*Nemalion elminthoides* (Velley) Batters	19	Native	New record
Rhodophyta	*Nitophyllum punctatum* (Stackhouse) Greville	1	Native	New record
Rhodophyta	*Osmundea hybrida* (A.P.de Candolle) K.W.Nam	1	Native	
Rhodophyta	*Osmundea oederi* (Gunnerus) G.Furnari	5	Native	New record
Rhodophyta	*Osmundea pinnatifida* (Hudson) Stackhouse	10	Native	
Rhodophyta	*Osmundea truncata* (Kützing) K.W.Nam & Maggs	23	Native	
Rhodophyta	*Palisada patentiramea* (Montagne) Cassano, Sentíes, Gil-Rodríguez & M.T.Fujii	2	Native	New record
Rhodophyta	*Peyssonnelia squamaria* (S.G.Gmelin) Decaisne ex J.Agardh	4	Native	
Rhodophyta	*Phyllophora crispa* (Hudson) P.S.Dixon	5	Native	New record
Rhodophyta	*Phyllophora gelidioides* P.Crouan & H.Crouan ex Karsakoff	1	Macaronesian endemism	
Rhodophyta	*Phymatolithon lenormandii* (Areschoug) Adey	1	Native	New record
Rhodophyta	*Platoma cyclocolpum* (Montagne) F.Schmitz	13	Native	New record
Rhodophyta	*Pleonosporium borreri* (Smith) Nägeli	15	Native	
Rhodophyta	*Plocamium cartilagineum* (Linnaeus) P.S.Dixon	13	Native	
Rhodophyta	*Polysiphonia ceramiiformis* P.Crouan & H.Crouan	1	Native	New record
Rhodophyta	*Polysiphonia stricta* (Mertens ex Dillwyn) Greville	1	Native	
Rhodophyta	Predaea feldmannii subsp. azorica Gabriel	7	Azorean endemism	
Rhodophyta	*Pterocladiella capillacea* (S.G.Gmelin) Santelices & Hommersand	44	Native	
Rhodophyta	*Pterothamnion crispum* (Ducluzeau) Nägeli	1	Native	
Rhodophyta	*Ptilothamnion pluma* (Dillwyn) Thuret	2	Uncertain	
Rhodophyta	*Rhodophyllis divaricata* (Stackhouse) Papenfuss	1	Native	New record
Rhodophyta	*Rhodymenia holmesii* Ardissone	7	Native	
Rhodophyta	*Rhodymenia pseudopalmata* (J.V.Lamouroux) P.C.Silva	12	Native	New record
Rhodophyta	*Schizymenia apoda* (J.Agardh) J.Agardh	3	Native	
Rhodophyta	*Scinaia interrupta* (A.P.de Candolle) M.J.Wynne	30	Native	
Rhodophyta	*Sphaerococcus coronopifolius* Stackhouse	7	Native	New record
Rhodophyta	*Spyridia filamentosa* (Wulfen) Harvey	3	Native	New record
Rhodophyta	*Stypopodium zonale* (J.V.Lamouroux) Papenfuss	2	Native	New record
Rhodophyta	*Symphyocladia marchantioides* (Harvey) Falkenberg	5	Introduced	
Rhodophyta	*Vertebrata fruticulosa* (Wulfen) Kuntze	12	Native	
Rhodophyta	*Vertebrata hypnoides* (Welwitsch) Kuntze	4	Uncertain	
Rhodophyta	*Vertebrata reptabunda* (Suhr) Díaz-Tapia & Maggs	5	Uncertain	
Rhodophyta	*Vertebrata tripinnata* (Harvey) Kuntze	2	Native	
Rhodophyta	*Xiphosiphonia pennata* (C.Agardh) Savoie & G.W.Saunders	1	Native	
Rhodophyta	*Xiphosiphonia pinnulata* (Kützing) Savoie & G.W.Saunders	2	Introduced	
Rhodophyta	*Yuzurua poiteaui* (J.V.Lamouroux) Martin-Lescanne	5	Native	New record

**Table 4.** Main taxonomic figures with information on the species origin and status.

Phyllum	Order	Family	Specimens Number	Total taxa	Total species	Native	Introduced	Uncertain	Macaronesian endemism	Azorean endemism	New record
Rhodophyta	17	41	1072	166	126	99	10	13	3	1	47
Chlorophyta	4	9	236	36	31	27	1	2	1		10
Ochrophyta	10	16	384	48	38	30	3	5			22
Total	31	66	1692	250	195	156	14	20	4	1	79
